# miRExpress: Analyzing high-throughput sequencing data for profiling microRNA expression

**DOI:** 10.1186/1471-2105-10-328

**Published:** 2009-10-12

**Authors:** Wei-Chi Wang, Feng-Mao Lin, Wen-Chi Chang, Kuan-Yu Lin, Hsien-Da Huang, Na-Sheng Lin

**Affiliations:** 1 Institute of Bioinformatics and Systems Biology, National Chiao Tung University, Hsin-Chu 300, Taiwan, Republic of China; 2 Institute of Biotechnology, National Cheng Kung University, Tainan 701, Taiwan, Republic of China; 3 Institute of Plant and Microbial Biology, Academia Sinica, Nankang, Taipei 11529, Taiwan, Republic of China; 4 Department of Biological Science and Technology, National Chiao Tung University, Hsin-Chu 300, Taiwan, Republic of China; 5 Institute of Tropical Plant Science, National Cheng Kung University, Tainan 701, Taiwan, Republic of China

## Abstract

**Background:**

MicroRNAs (miRNAs), small non-coding RNAs of 19 to 25 nt, play important roles in gene regulation in both animals and plants. In the last few years, the oligonucleotide microarray is one high-throughput and robust method for detecting miRNA expression. However, the approach is restricted to detecting the expression of known miRNAs. Second-generation sequencing is an inexpensive and high-throughput sequencing method. This new method is a promising tool with high sensitivity and specificity and can be used to measure the abundance of small-RNA sequences in a sample. Hence, the expression profiling of miRNAs can involve use of sequencing rather than an oligonucleotide array. Additionally, this method can be adopted to discover novel miRNAs.

**Results:**

This work presents a systematic approach, miRExpress, for extracting miRNA expression profiles from sequencing reads obtained by second-generation sequencing technology. A stand-alone software package is implemented for generating miRNA expression profiles from high-throughput sequencing of RNA without the need for sequenced genomes. The software is also a database-supported, efficient and flexible tool for investigating miRNA regulation. Moreover, we demonstrate the utility of miRExpress in extracting miRNA expression profiles from two Illumina data sets constructed for the human and a plant species.

**Conclusion:**

We develop miRExpress, which is a database-supported, efficient and flexible tool for detecting miRNA expression profile. The analysis of two Illumina data sets constructed from human and plant demonstrate the effectiveness of miRExpress to obtain miRNA expression profiles and show the usability in finding novel miRNAs.

## Background

MicroRNAs (miRNAs), small non-coding RNAs of 19 to 25 nt, play important roles in gene regulation in both animals and plants. Generally, miRNAs hybridize to the 3'-untranslated region of mRNA to downregulate gene expression or to induce the cleavage of mRNA and can fully hybridize to the transcripts of target genes [1]. Previous studies have suggested that miRNAs are strongly associated with various cancers and are considered tumor suppressors or oncogenes [2].

In the last few years, high-throughput and robust approaches for monitoring the expression of miRNAs have been used to understand how miRNAs are differentially expressed under various conditions. The oligonucleotide microarray is one method for detecting miRNA expression [3,4]. This approach involves the design of probes based on known miRNAs that are collected in miRBase [5] for miRNA expression profiling studies [6,7]. However, the approach is restricted to detecting the expression of known miRNAs.

Second-generation sequencing is an inexpensive and high-throughput sequencing method. This new method is a promising tool with high sensitivity and specificity and can be used to measure the abundance of small-RNA sequences in a sample. Hence, the expression profiling of miRNAs can involve use of sequencing rather than an oligonucleotide array [8-15]. Additionally, this method can be adopted to discover novel miRNAs. Numerous investigations have applied second-generation sequencing for discovering and profiling miRNA in various species. Using small-RNA sequencing, Glazov *et al*. identified 449 novel miRNAs in chicken embryo and 32 differentially expressed known chicken miRNAs in three embryonic small RNA libraries [9]. Morin *et al*. discovered 104 novel miRNAs in human embryonic stem cells (hESCs) and 171 differentially expressed known human miRNAs in two developmental states [11]. Stark *et al*. identified 41 novel miRNA genes in *Drosophila *and used these sequencing reads to validate 28 of the genes [13]. Sunkar *et al*. identified 23 novel miRNAs in rice and found 82 differentially expressed miRNAs in three libraries [14].

These sequencing schemes can generate millions of short sequences. Profiling miRNA expression levels with second-generation sequencing technologies involves aligning sequences to those in a genome. However, aligning millions of sequences to those in a genome is less efficient than aligning to sequences of known miRNAs. Several previous studies apply next-generation sequencing to profile miRNA expression through aligning sequences against genomic sequence [9,11]. In their work, blat [16] or BLAST [17] are utilized to map sequencing reads of small-RNAs to genomic sequence and the sequences of known miRNAs. However, the alignment software, BLAST and blat, cannot efficiently handle a huge scale of short sequences. To solve this problem, other programs, such as RMAP [18], SeqMap[19], ZOOM [20], Maq [21] and SOAP [22], are developed for analyzing numerous short sequences and map millions of short sequences to genomic sequences. However, these software cannot achieve their capability when the genomic sequences are not available. In detecting miRNA expression, aligning millions of sequencing reads to genomic sequences can be alternatively replaced by aligning these sequencing reads against the sequences of known miRNAs. Consequently, a tool designed for constructing miRNA expression profile through directly aligning millions of short sequences with the sequences of known miRNAs is certainly needed.

This work presents a systematic method, miRExpress, for extracting miRNA expression profiles from sequencing reads generated by second-generation sequencing. miRExpress is the first stand-alone package that contains miRNA information from miRBase [5] and efficiently reveals miRNA expression profiles by aligning sequencing reads against the sequences of known miRNAs. This approach can be used to determine miRNA expression profiles when genomic sequences are unavailable and can greatly reduce the time spent aligning sequencing reads and genomic sequences. Furthermore, miRExpress can be used to find novel miRNA candidates by aligning reads with sequences of known miRNAs of various species. We used miRExpress to extract the miRNA expression profiles from two Illumina data sets constructed for the human and a plant species to demonstrate the utility of miRExpress. One data set is a publicly available Illumina sequencing data set of two developmental states of hESCs [11], and the other is a data set we generated in this work using Illumina sequencing for three inoculations of *Arabidopsis*.

## Implementation

**Figure 1:**
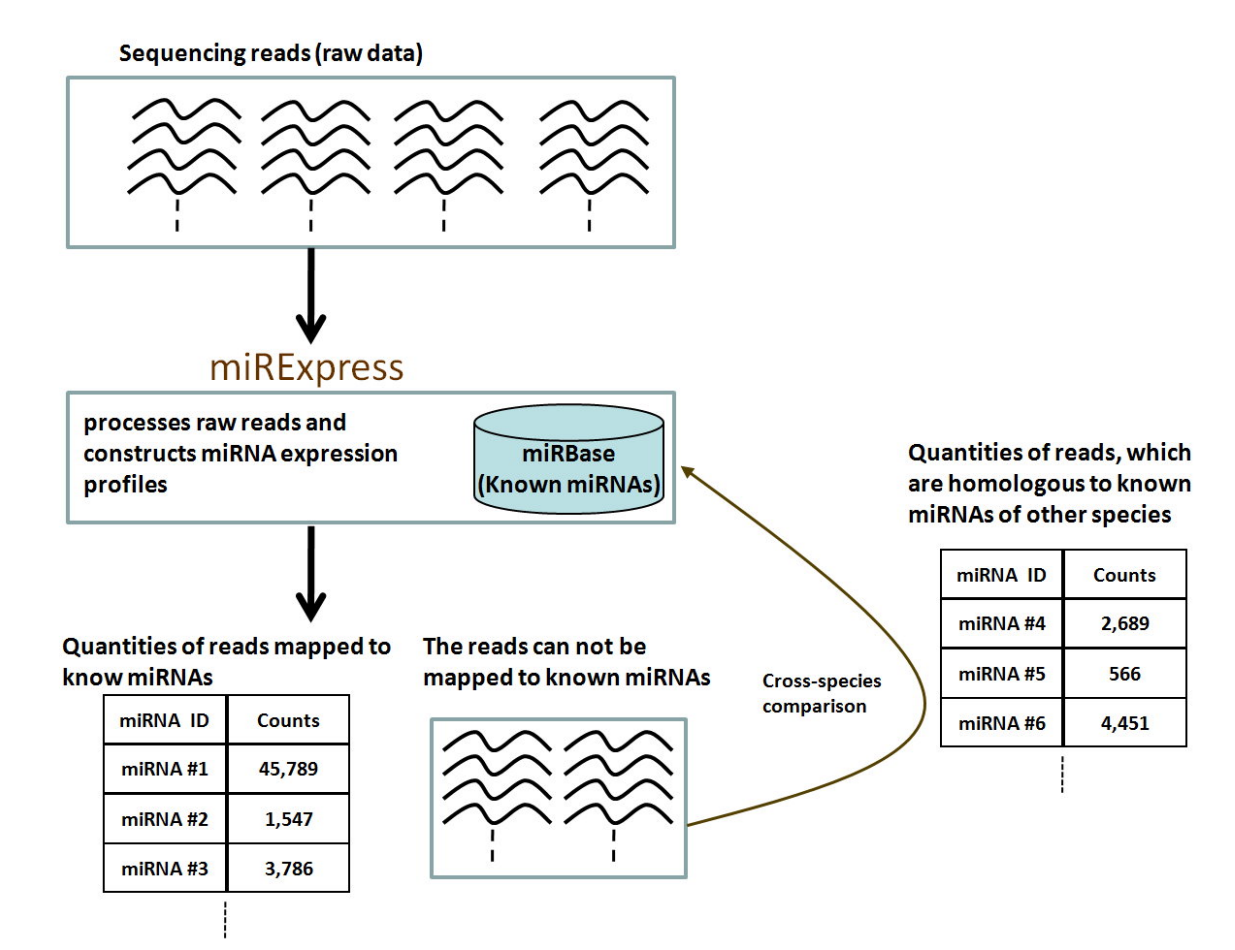
**System flow of construction of miRNA expression profiles**. miRExpress can accept second-generation sequencing data and generate miRNA expression profiles by aligning sequences of known miRNAs.

**Figure 2:**
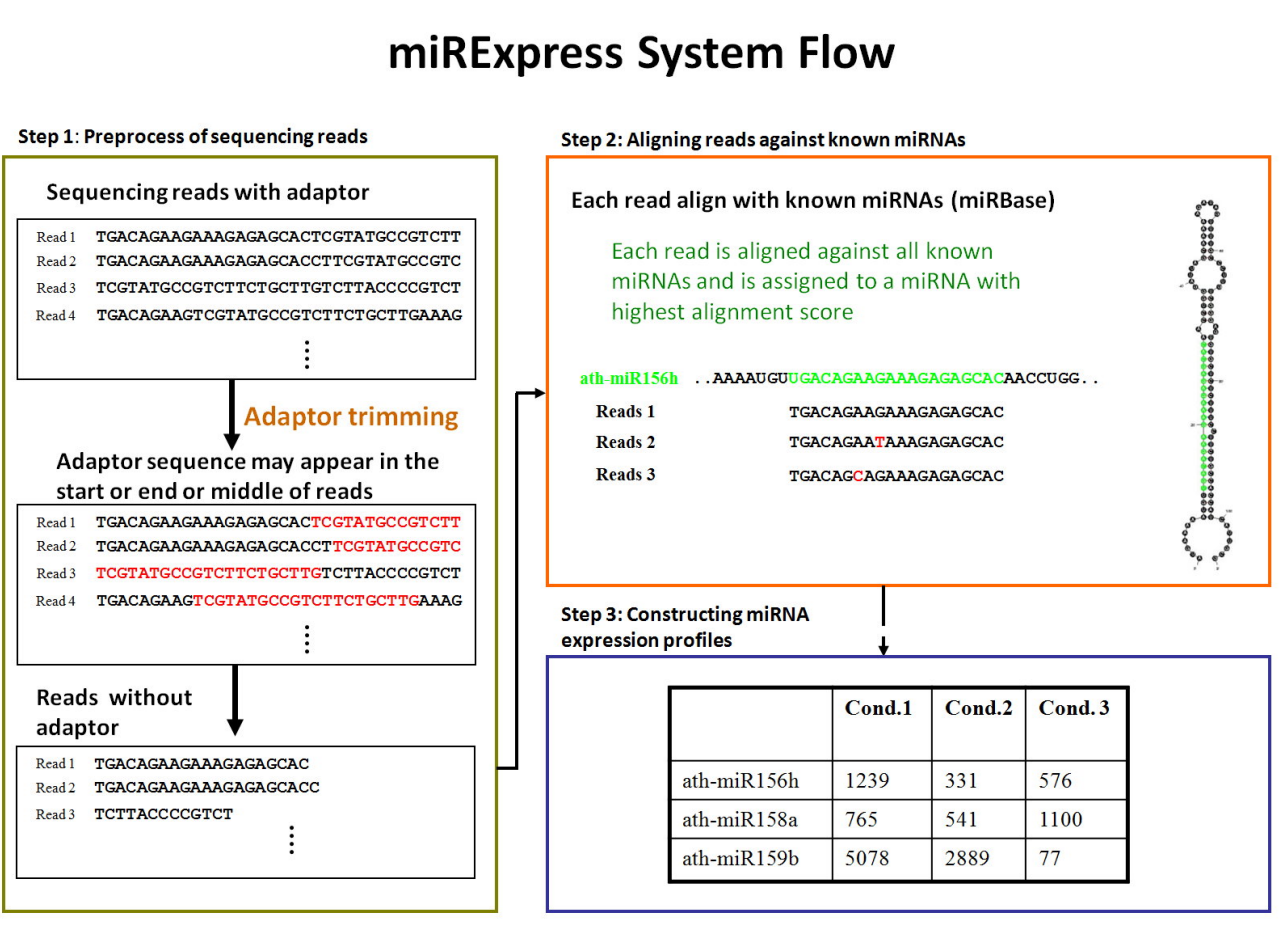
**miRExpress system flow has three steps: 1) Preprocessing original Illumina reads; 2) aligning reads with sequences of known miRNAs and 3) constructing miRNA expression profiles**.

Figure 1 simplifies the system flow of the construction of miRNA expression profiles with use of miRExpress, which accepts second-generation sequencing data as inputs and constructs miRNA expression profiles by aligning sequences with those of known miRNAs. The second-generation sequencing input data may define one or more experimental conditions. The process by which miRExpress constructs the miRNA expression profiles consists of three steps, as presented in Figure 2. The first step is the preprocessing of raw data obtained by second-generation sequencing. The second step is the alignment of all sequencing reads against those of known mature miRNAs. The third step is the construction of miRNA expression profiles from the results of the alignment.

In the first step, miRExpress merges the identical reads into a unique read and counts each unique read. Then, each unique read is checked to determine whether it contains a full or a partial adaptor sequence. In checking the full adaptor sequence, if the adaptor sequence is in the middle and at the beginning of the read, then the read is removed. If the adaptor sequence is at the end of the read, then the adaptor sequence is trimmed from the read sequence. In checking the partial adaptor sequence, the last bases of the 5' adaptor are used as a probe to match the first bases of the reads. The first bases of the 3' adaptor are used as probes to match the last bases of the reads. If the sequence identities of the matched regions are greater than 70%, these regions are eliminated.

In the second step, each read is aligned with the sequences of known mature miRNAs. The information for known miRNAs was obtained from miRBase (Release 12.0). In miRExpress, the same sequences from different miRNAs are analyzed as a single sequence. For example, ath-miR157a, ath-miR157b and ath-miR157c have the same sequence. The proposed alignment algorithm is based on the Smith-Waterman algorithm and implemented by following Single Instruction Multiple Data (SIMD) instructions [23]. When miRExpress is executed on a PC machine with an SSE3 instruction set, it can compare one miRNA with eight sequencing reads simultaneously. However, the proposed software is multiple-processor ready. For example, miRExpress can compare one miRNA with 64 sequencing reads simultaneously on a computer with eight processing cores. Another advantage is the use of a lookup table for scoring: the proposed algorithm can feasibly change the score or penalty for every pair of nucleotides as easily as when changing the match score and mismatch of only one nucleotide pair.

In the third step, miRNA expression profiles are constructed by computing the sum of read counts for each miRNA according to the alignment criteria (e.g., the length of the read equals the length of the miRNA sequence and the identity of the alignment is 100%). Users can set the cutoff of alignment identity based on their requirements when using miRExpress.

Previous studies suggest that RNA editing occurred in miRNAs can affect their interactions to targets and regulate the gene expression [24-27]. The sequence reads with high similarity to known miRNAs are valuable for further analysis of RNA editing and mutations occurred in miRNAs. Hence, we also provide a function in miRExpress which can return the sequence reads that are highly similar to known miRNAs including the nucleotide mismatch information.

## Results

### Evaluation of Illumina reads

In this work, we used two data sets generated using second-generation sequencing to evaluate the capability and effectiveness of miRExpress. The first data set was constructed in this work. The Illumina sequencing reads were generated from three inoculations of *Arabidopsis thaliana *(ecotype Columbia-0). For each inoculation, *A. thaliana *plants were inoculated with water (Mock), *Bamboo mosaic virus *(BaMV) alone, or co-inoculated with BSF4 satellite BaMV (satBaMV) (BaMV + satBaMV). SatBaMV is a subviral agent and depends on BaMV for replication, encapsidation and movement. BaMV and satBaMV were prepared as described [28]. RNA extracted from three inoculated leaves of *Arabidopsis *was used for sequencing with the Illumina Genome Analyzer System following the instruction from FASTERIS which is a biotechnology company headquartered in Geneva, Switzerland http://www.fasteris.com/.

The other data set consisted of Illumina sequencing reads taken from publicly available data generated by Morin *et al*. [11]. The sequencing of small-RNA libraries yielded 6,147,718 and 6,014,187 37-nt unfiltered sequencing reads from hESCs (before differentiation) and human cell aggregates called embryoid bodies (hEBs, after differentiation), respectively. The hESC sample was harvested by use of trypsin under non-adherent conditions that induced hESCs to differentiate into cells in all three germ layers and form hEBs. We analyzed the data set to elucidate the roles of miRNAs in the two developmental states (before and after differentiation) of hESCs.

### miRNA expression profiles in Arabidopsis

*Arabidopsis *miRNA expression profiles were constructed under three experimental conditions based on an alignment identity of 100% and a length of reads that equals the length of miRNA sequences. Table 1 presents the top 20 known *Arabidopsis *miRNAs expressed under three experimental conditions (full data are available in additional file 1). For example, the miRNA with the top expression level (read count) is ath-miR158a, with 178,820, 105,649 and 59,682 read counts for Mock, BaMV and BaMV+satBaMV treatment, respectively. The results suggest that ath-miR158a might play critical roles in BaMV infection mechanism in *Arabidopsis*. Consequently, it demonstrates that miRExpress facilitates the detection of miRNA expressions from huge data generated by next-generation sequencing of small-RNAs.

**Table 1:** Top 20 expressed miRNAs in Arabidopsis obtained by miRExpress.

miRNA ID	Mock	BaMV	BaMV+satBaMV (count)
ath-miR158a	178,820	105,649	59,682
ath-miR157(a, b, c)	103,140	46,113	17,695
ath-MIR166(a, b, c, d, e, f. g)	92,819	67,641	48,087
ath-miR156(a, b, c, d, e, f)	63,539	36,906	24,926
ath-miR167(a, b)	29,899	30,542	7,712
ath-miR168(a, b)	8,630	5,533	2,599
ath-miR172(a, b)	6,805	5,438	898
ath-miR391	6,139	6,466	2,625
ath-miR173	4,523	2,973	545
ath-miR161.2	2,687	1,112	318
ath-miR164c	1,178	1,818	501
ath-miR164(a, b)	1,155	1,797	479
ath-miR165(a, b)	1,083	1,125	740
ath-miR390(a, b)	936	1,152	533
ath-miR159a	910	744	230
ath-miR408	829	658	265
ath-miR163	720	766	282
ath-miR822	470	207	95
ath-miR396a	448	312	62
ath-miR167d	446	361	67

### miRNA expression profiles in human

The miRNA expression profiles of hESCs were constructed under two experimental conditions, hESCs and hEBs, based on an alignment identity of 100% and read lengths that equaled the length of miRNAs. Table 2 presents the 20 known human miRNAs expressed under two experimental conditions (full data are available in additional file 2). For example, the expression levels (read counts) for hsa-mir-25-3p are 24,268 and 15,875 for hESCs and hEBs, respectively. We demonstrated that the expression profiles of known miRNAs discovered by miRExpress can be comparable with the results provided by Morin et al.

**Table 2:** Top twenty miRNAs expressed in human embryonic stem cells (hESCs) and human embryoid bodies (hEBs) obtained by miRExpress.

miRNA ID	Pre-mRNA arm (5-p or 3-p)	hESCs	hEBs (count)
hsa-mir-25	3-p	24268	15875
hsa-mir-221	3-p	16275	8716
hsa-mir-302b	3-p	15169	8855
hsa-let-7a	5-p	11902	2951
hsa-mir-423	5-p	9844	5538
hsa-mir-302d	3-p	8599	5047
hsa-mir-1	3-p	7421	4051
hsa-mir-320	3-p	5967	2978
hsa-mir-363	3-p	5775	17912
hsa-mir-302a	3-p	5239	3237
hsa-mir-26a	5-p	4892	8530
hsa-mir-744	5-p	4166	1516
hsa-mir-130a	3-p	2334	4798
hsa-mir-340	5-p	2247	7198
hsa-let-7f	5-p	2004	1281
hsa-mir-372	3-p	1388	13653
hsa-mir-423	3-p	1225	560
hsa-mir-331	3-p	1129	434
hsa-mir-199a	3-p	1110	13163
hsa-mir-129	3-p	946	449

### Discovery of novel human miRNAs by aligning reads to mammalian but not human miRNAs

**Figure 3:**
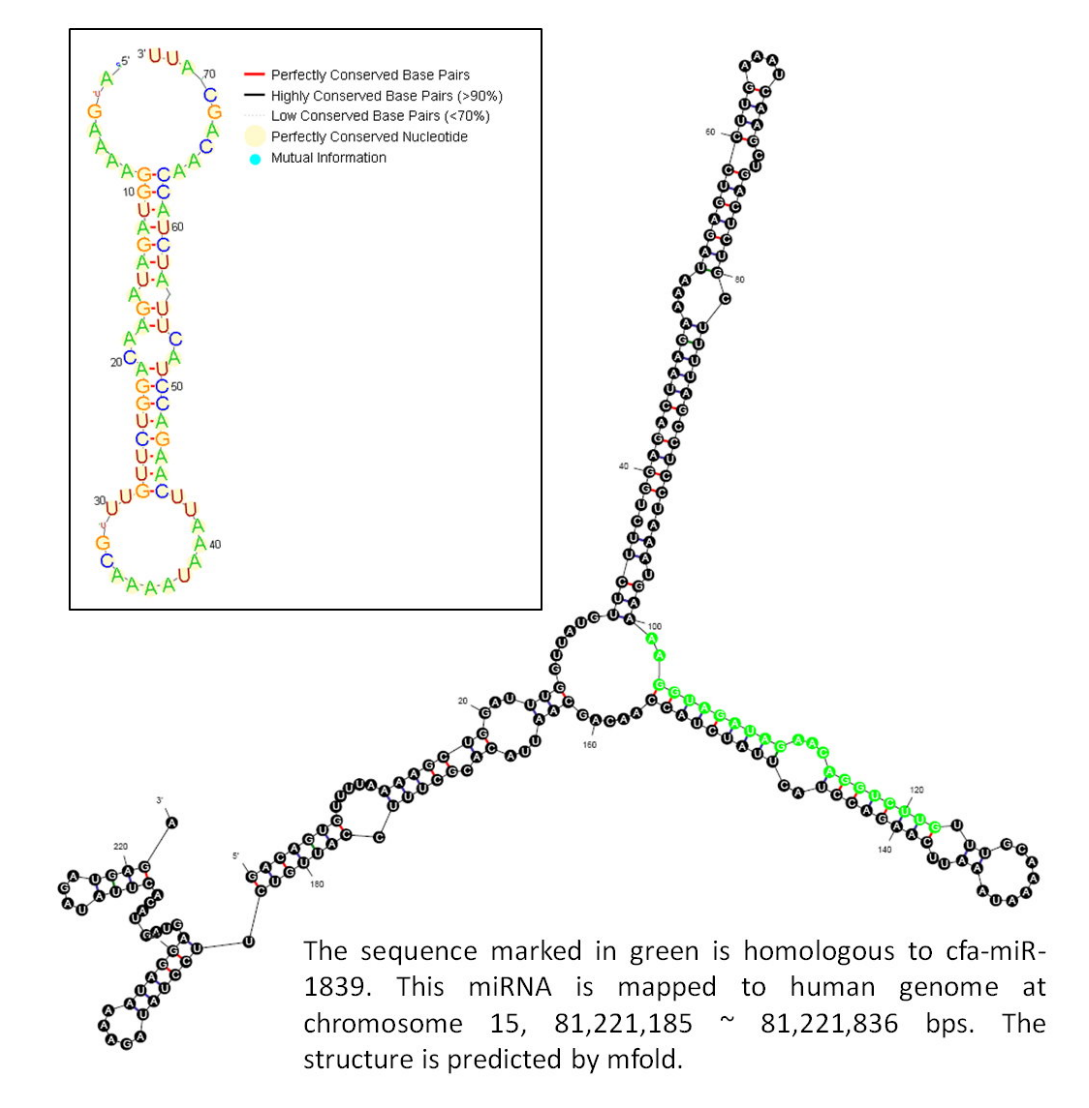
**The precursor of hsa-mir-putative-1**.

**Figure 4:**
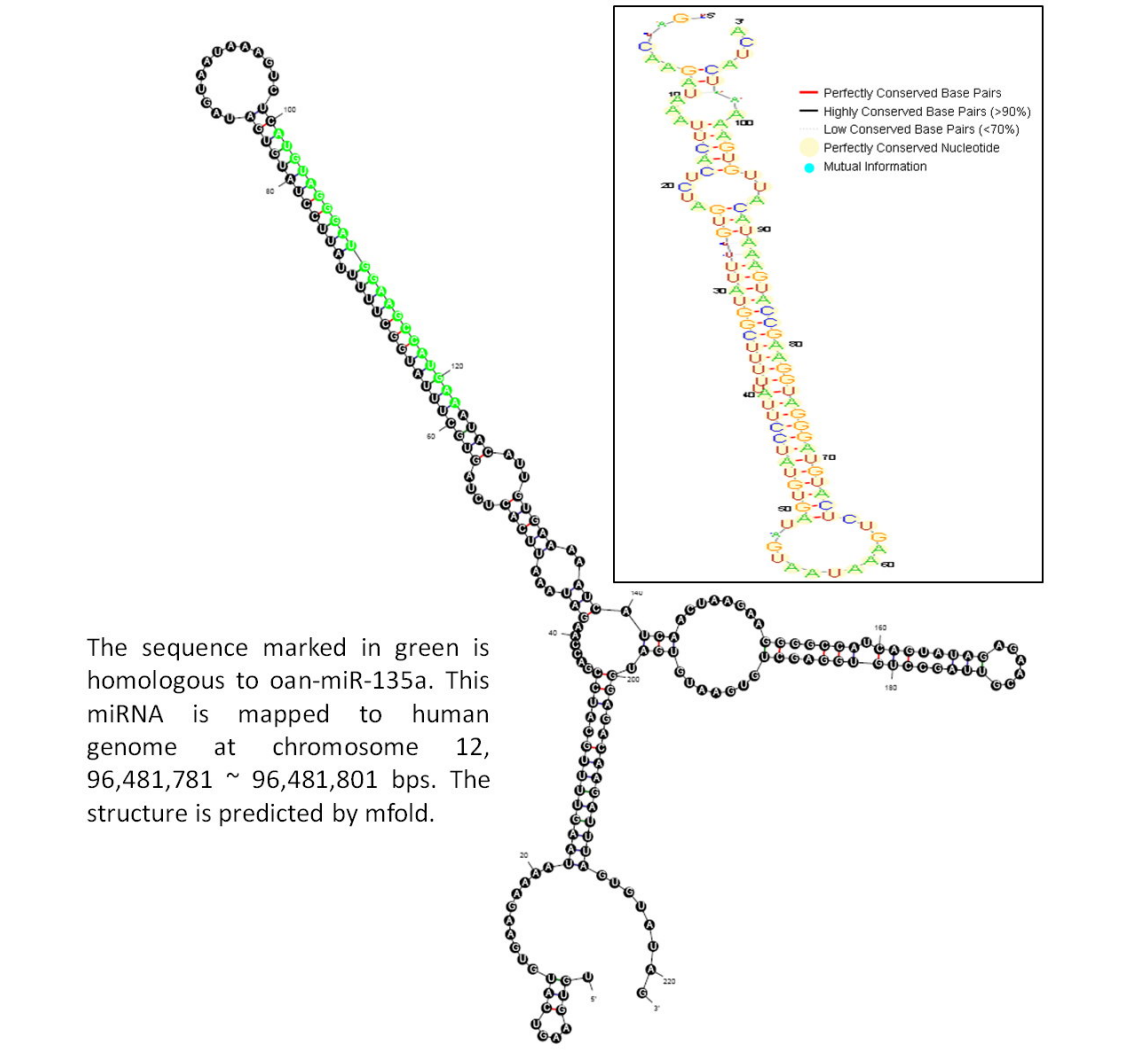
**The precursor of hsa-mir-putative-2**.

In hESCs and hEBs, 40 and 39 novel miRNAs, respectively, were detected by aligning reads that cannot be mapped to known human miRNAs for all mammals, excluding human miRNAs. The alignment criteria are an alignment identity of 100% and read lengths that equal the length of miRNAs. Table 3 gives the top 10 expressed miRNAs belong to different species (full data are available in additional file 3). For example, the most abundant sequencing reads matched to cfa-miR-1839 has 9,332 read counts in hESCs and 8,007 in hEBs. In order to assess the potential miRNA precursor of the putative miRNA, 100 nt 5'-flank and 100 nt 3'-flank are extracted from the matched region after aligning the putative miRNA against the human genomic sequence. We use mfold [29], which is a software for folding secondary structure from a RNA sequence, to obtain putative RNA secondary structures of miRNA precursor. Figure 3 shows the structure of a human putative miRNA (hsa-mir-putative-1), which is homologous to cfa-miR-1839. Another human putative miRNA (hsa-mir-putative-2) homologous to oan-miR-135a is presented in Figure 4. Moreover, we obtain the cross-species sequence information from UCSC Genome Browser [30] to observe the conservation of putative miRNAs. From the RNA structural logo generated by RNALogo [31], the precursor of hsa-mir-putative-1 shows that it is highly conserved among dog, human, mouse and rat. Similarly, the precursor of hsa-mir-putative-2 is well conserved among six different species, i.e., platypus, opossum, human, mouse, chicken and lizard. Consequently, miRExpress can produce abundant sequencing reads of small-RNAs for discovery of novel miRNAs.

**Table 3:** Top 10 expressed miRNAs in human embryonic stem cells (hESCs) and human embryoid bodies (hEBs) detected by aligning reads with sequences from mammalian (but not human) miRNAs.

miRNA ID	hESCs (count)	hEBs (count)
cfa-miR-1839	9,332	8,007
oan-miR-135a	299	336
rno-miR-1	69	37
mmu-miR-452	58	52
oan-miR-365*	48	19
mmu-miR-302a*	45	5
rno-miR-135a*, cfa-miR-135	42	68
oan-miR-92b	38	201
oan-miR-301*	17	30
oan-miR-130c	11	37

## Discussion

### Experimental errors associated with Illumina sequencing

In human and *Arabidopsis *data sets, the middle 0.12%~0.87% of the sequencing reads have an adaptor sequence (Table 4). The location of an adaptor in the middle of the reads means that the start location of the full-length adaptor in the read is not the start of the read. For example, the read sequence "AAGCCAAGGTCGTATGCCGTCTTCTGCTTGGAAAAA" contains the full-length adaptor from 10 to 30. This condition reveals that Illumina sequencing has a few experimental errors, but these do not affect further analysis.

**Table 4:** Number of sequencing reads that have adaptors in the middle.

Experiment	Total reads	Adaptor in the middle of reads
hESC (human)	6,147,718	33,981
hB (human)	6,014,187	18,546
Mock(Arabidopsis)	5,265,076	46,110
BaMV(Arabidopsis)	4,039,593	22,298
BaMV+satBaMV(Arabidopsis)	2,865,495	3,542

### Comparing the results of the application of a trimming adaptor to Arabidopsis obtained by miRExpress with those obtained by other schemes

To confirm the workability of the trimming adaptor method in miRExpress, we compared the use of a trimming adaptor with miRExpress to that with another method, FASTERIS. Use of FASTERIS involves three steps. First, the adapter sequence is employed as a probe, which allows for exact match inserts to be identified. Second, if no adapter sequence is found, the last bases of the sequencing reads are probed successively with use of the first bases of the adapter (minimum five bases) until a match is found, identifying inserts in up to 30 bases. Third, the remaining reads are searched to identify non-exact matches of the adapter. The first four bases of the adapter are used as a probe. The following properties are adopted to determine the presence of the adapter. Seventy-five percent of them must be identical to the adapter sequence. The read number distribution is consistent with the read length distribution (Additional file 4). The correlation coefficients of the read number distribution for the miRExpress and FASTERIS methods under Mock, BaMV and BaMV+satBaMV conditions are 0.999, 0.998 and 0.996, respectively. The correlation coefficients of the read count distribution under Mock, BaMV and BaMV+satBaMV conditions are 0.997, 0.996 and 0.986, respectively (Additional file 5). The high correlation coefficient indicates that the two methods of trimming the adaptor sequence yield the same results for different lengths of reads. Accordingly, the results of applying the trimming adopter sequences with these two methods are mutually consistent.

### Comparing miRNA expression profiles produced by miRExpress and alignment of reads to genome sequences in Arabidopsis

**Figure 5:**
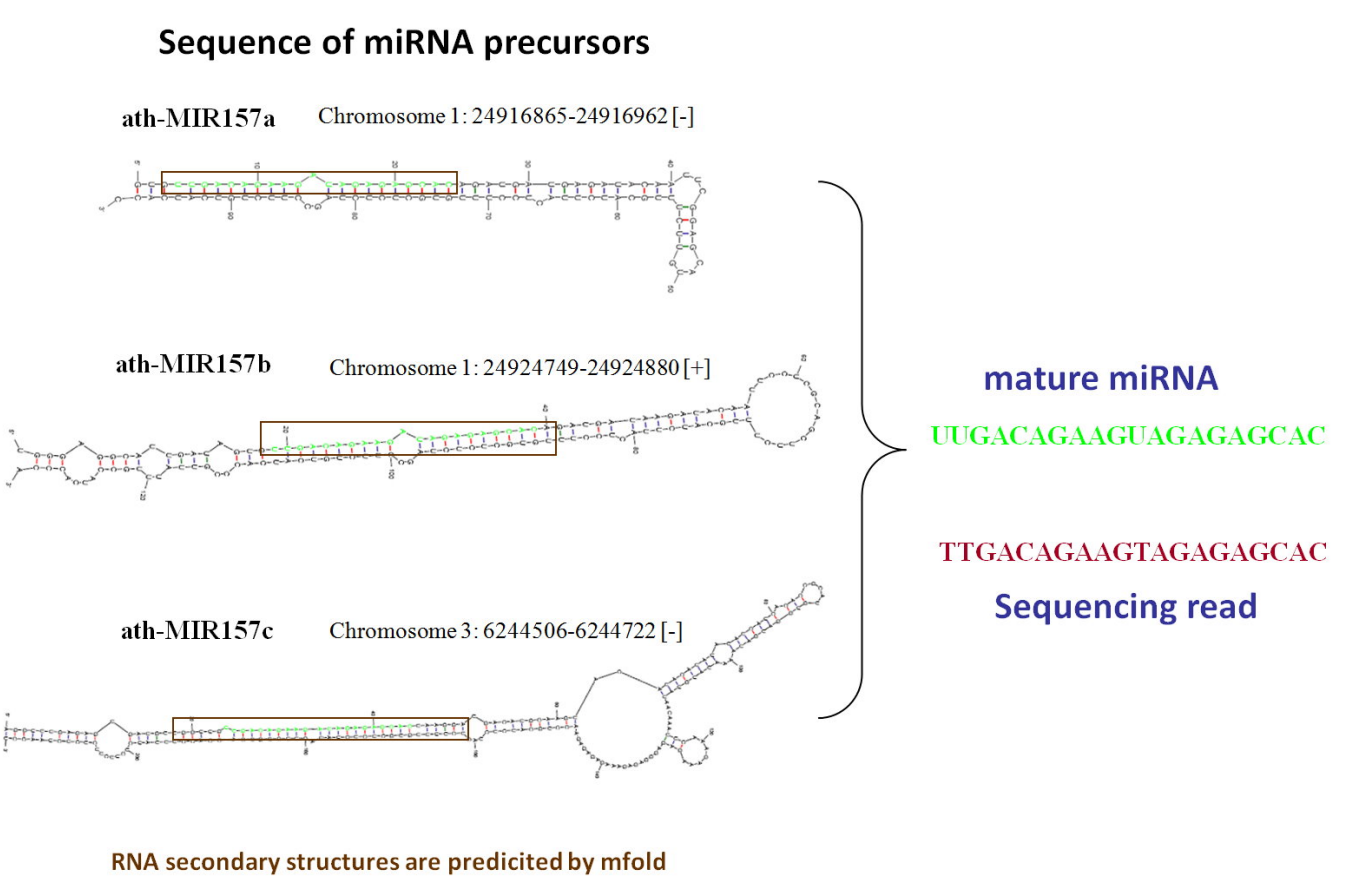
**ath-MIR 157a, ath-MIR 157b and ath-MIR 157c are located in various genomic locations but have the same mature sequences**.

A comparison of miRNA expression profiles generated with miRExpress and the alignment of sequence reads to genome sequences of *Arabidopsis *suggests that the construction of miRNA expression profiles by aligning sequence reads with those of known miRNAs is equivalent to aligning the reads to genome sequences. Table 5 shows the miRNAs with the top 20 expression levels under Mock, BaMV and BaMV+satBaMV treatment by both methods. Additionally, aligning reads with miRNA sequences can reduce the time required to profile the expression of miRNAs to less than that required for aligning reads with genome sequences (all data are available in additional file 6). These two miRNA expression profiles are both constructed according to the criteria that the alignment identity must be 100% and the length of the reads must equal the length of miRNAs, such that miRNAs that have the same sequence are considered as a single sequence in the detection of the expression levels. For example, ath-miR157a, ath-miR157b and ath-miR157c have the same sequence but are generated from different chromosome loci. These miRNAs are regarded as one sequence in the determination of their expression levels because when reads can perfectly match these miRNAs, the miRNAs to which they belong cannot be identified (Figure 5).

**Table 5:** Top 20 expressed miRNAs in Arabidopsis under Mock, BaMV and BaMV+BSF4 conditions obtained with miRExpress and BLAST search of genomic sequences.

	miRExpress			BLAST		
**microRNA ID**	**Mock**	**BaMV**	**BaMV+ satBaMV**	**Mock**	**BaMV**	**BaMV+satBaMV (count)**

ath-miR158a	178,820	105,649	59,682	178,820	105,649	59,682
ath-miR157 (a, b, c)	103,140	46,113	17,695	103,140	46,113	17,695
ath-miR166 (a, b, c, d, e, f. g)	92,819	67,641	48,087	92,819	67,641	48,087
ath-miR156 (a, b, c, d, e, f)	63,539	36,906	24,926	63,539	36,906	24,926
ath-miR167(a, b)	29,899	30,542	7,712	29,899	30,542	7,712
ath-miR168(a, b)	8,630	5,533	2,599	8,630	5,533	2,599
ath-miR172(a, b)	6,805	5,438	898	6,805	5,438	898
ath-miR391	6,139	6,466	2,625	6,139	6,466	2,625
ath-miR173	4,523	2,973	545	4,523	2,973	545
ath-miR161.2	2,687	1,112	318	2,687	1,112	318
ath-miR164c	1,178	1,818	501	1,178	1,818	501
ath-miR164(a, b)	1,155	1,797	479	1,155	1,797	479
ath-miR165(a, b)	1,083	1,125	740	1,083	1,125	740
ath-miR390(a, b)	936	1,152	533	936	1,152	533
ath-miR159a	910	744	230	910	744	230
ath-miR408	829	658	265	829	658	265
ath-miR163	720	766	282	720	766	282
ath-miR822	470	207	95	470	207	95
ath-miR396a	448	312	62	448	312	62
ath-miR167d	446	361	67	446	361	67

### Comparing miRNA expression profiles generated by miRExpress and method of Morin *et al*

**Figure 6:**
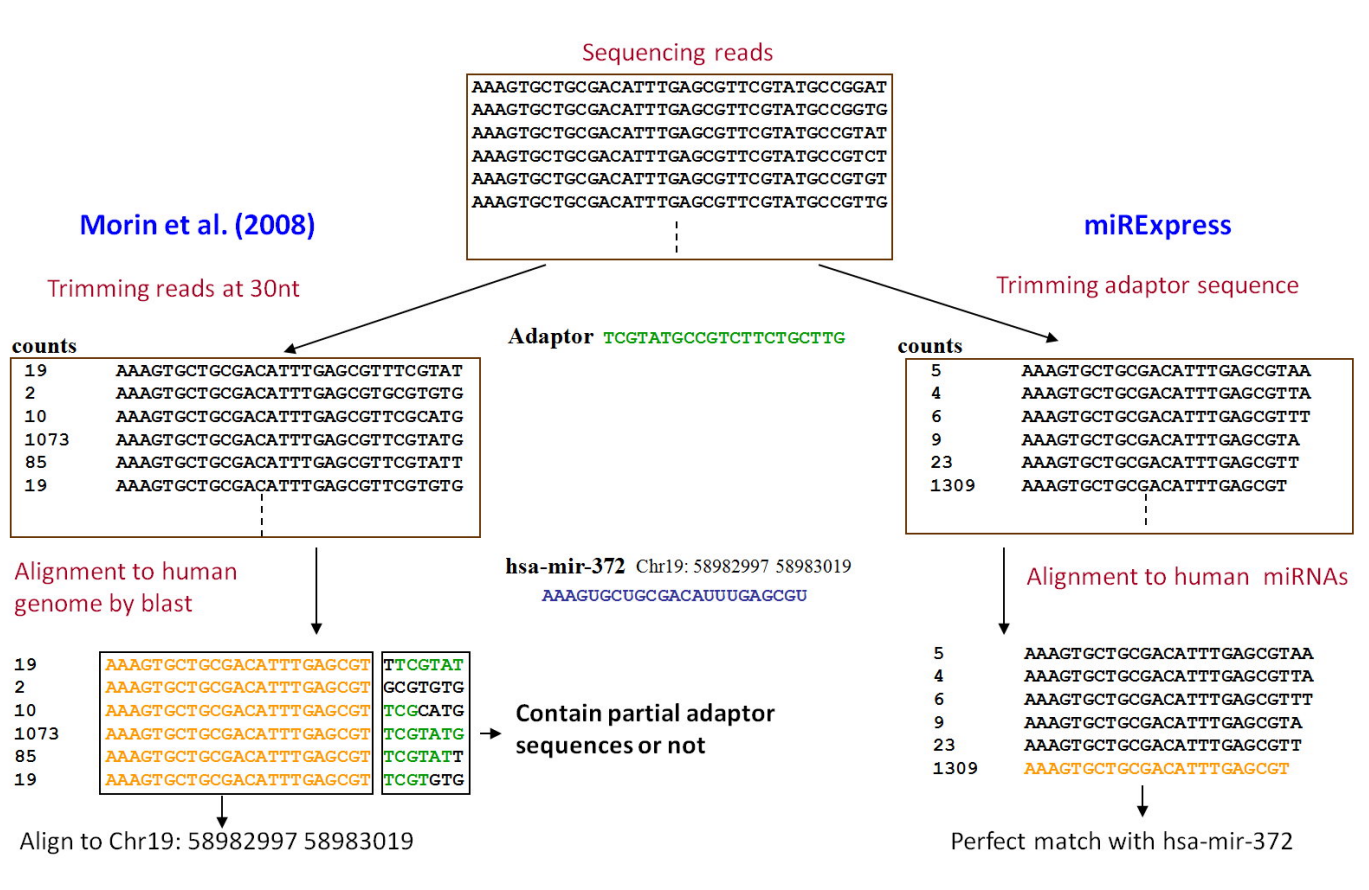
**Trimming adaptor sequences by Morin *et al*. and miRExpress methods yields different detected miRNA expression levels**.

A comparison of miRNA expression profiles produced by miRExpress and Morin *et al*. for the human species suggests that different adaptor trimming schemes might result in different miRNA expression levels. Among 20 expressed miRNAs, the expression levels of hsa-mir-25, hsa-mir-221, hsa-mir-302b, hsa-mir-363, hsa-mir-372, hsa-mir-199a, hsa-mir-302d, hsa-mir-26a, hsa-mir-320, hsa-mir-744, hsa-mir-152 and hsa-let-7e in the study of Morin *et al*. exceed those obtained with miRExpress, but the levels of hsa-mir-423, hsa-let-7a, hsa-mir-1, hsa-mir-340, hsa-mir-302a, hsa-mir-130a, hsa-let-7f and hsa-mir-122 in the work by Morin *et al*. are lower than those obtained from miRExpress (Table 6) (full data are available in additional file 7). The method of trimming the adaptor sequence is largely responsible for the difference in miRNA levels detected. Morin *et al*. firstly trimmed all reads at 30 nt. The authors then aligned these reads against the genome and identified the longest alignment for every read. Finally, they determined whether the alignment region of read contained a 3' adaptor. With miRExpress, the trimming adaptor sequence is completed before reads are aligned to miRNA sequences and reads that can perfectly match miRNAs are found. For example, in detecting hsa-mir-372 expression levels, Morin *et al*. trimmed reads at 30 nt and aligned them with the human genome. The 1~23 bases of some reads were mapped to the hsa-mir-372 chromosome location. The 24~30 bases of these reads were trimmed by the Morin's methods, and the counts of these reads were summed to represent this miRNA expression level. However, the 24~30 bases of some of these reads are not partial adaptor sequences (Figure 6). For instance, the read sequence "AAAGTGCTGCGACATTTGAGCGTGCGTGTG" has the same sequence from bases 1 to 23 as that of mir-372, but bases 24~30 of its sequence "GCGTGCG" do not constitute a part of the adaptor sequence "TCGTATGCCGTCTTCTGCTTG". With miRExpress, after the adaptor sequence is trimmed and aligned with miRNA sequences, the perfectly matched read is determined. Therefore, we recommend that the analytical results of miRExpress are closer to the truth than the results generated by Morin's methods. Figure 6 clearly demonstrates that the method of Morin *et al*. but not miRExpress retains some reads whose 24~30 bases do not form a partial adaptor sequence. Accordingly, the detected expression level of hsa-mir-372 differs between the two methods.

**Table 6:** Twenty miRNAs expressed in human embryonic stem cells (hESCs) and human embryoid bodies (hEBs) obtained by miRExpress and Morin *et al*. methods.

		Morin *et al*.		miRExpress	
**miRNA ID**	**Pre-miRNA arm (5-p or 3-p)**	**hESCs**	**hEBs**	**hESCs**	**hEBs (count)**

hsa-mir-25	3-p	24268	15875	23508	15206
hsa-mir-221	3-p	16275	8716	13438	7134
hsa-mir-302b	3-p	15169	8855	12101	6302
hsa-let-7a	5-p	11902	2951	14865	3565
hsa-mir-423	5-p	9844	5538	11221	6424
hsa-mir-302d	3-p	8599	5047	7931	4597
hsa-mir-1	3-p	7421	4051	8576	4713
hsa-mir-320	3-p	5967	2978	4277	2118
hsa-mir-363	3-p	5775	17912	3228	8723
hsa-mir-302a	3-p	5239	3237	6045	3523
hsa-mir-26a	5-p	4892	8530	2887	3631
hsa-mir-744	5-p	4166	1516	2991	900
hsa-mir-130a	3-p	2334	4798	2370	4845
hsa-mir-340	5-p	2247	7198	2287	7251
hsa-let-7f	5-p	2004	1281	2427	1766
hsa-mir-372	3-p	1388	13653	1309	12922
hsa-mir-423	3-p	1225	560	1002	443
hsa-mir-331	3-p	1129	434	776	235
hsa-mir-199a	3-p	1110	13163	875	10214
hsa-mir-129	3-p	946	449	992	478

## Conclusion

This work develops miRExpress, which is a database-supported, efficient and flexible tool for detecting miRNA expression profile. All previous programs are designed based on mapping high-throughput sequencing reads to genomic sequence. However, miRExpress is the first design for detecting miRNA expression profile from next-generation sequencing reads of small-RNAs without the need of sequenced genomes. The expression profiles of miRNAs can be monitored if the sequenced genomes are not available. Since miRExpress does not align the sequencing reads against genomic sequences, it can greatly reduce the computational time required for the analysis (See additional file 8). Notwithstanding, similar concepts have been demonstrated in prior studies [32-34], none of them implements user-friendly and rapid analysis software for miRNAs expression profiling. Besides, miRExpress produces abundant sequencing reads, which can be served as basis to discover novel miRNAs, by aligning the sequencing reads to miRNAs in other species. However, people may argue that using known miRNAs from multiple species might miss some novel miRNAs that are yet to be discovered in any species. To address this issue, the highly expressed reads, which cannot be annotated as the expressed evidences of known miRNAs, are listed in miRExpress output pages. The listed sequencing data can be mapped to the genomic sequences for further analysis. Actually, we plan to extend this function in miRExpress in the near future. Moreover, the analysis of two Illumina data sets constructed from human and plant demonstrate the effectiveness of miRExpress to obtain miRNA expression profiles and show the usability in finding novel miRNAs.

## Availability and requirements

miRExpress software was written in C++ programming language and can be executed in 32 or 64 bit Linux machine. The software can be freely downloaded at http://miRExpress.mbc.nctu.edu.tw.

## Authors' contributions

HDH and NSL conceptualized the project. WCW and FML designed and built the database and web interface. WCW, FML, WCC, and KYL performed data analysis. NSL and KYL generate Illumina sequencing reads from *Arabidopsis thaliana*. HDH, WCW and WCC wrote the draft. All authors tested the database and interfaces. All authors read and approved the final manuscript.

## Supplementary Material

Additional file 1 **miRNA expressions profile in Arabidopsis**. Arabidopsis miRNA expression profile in three experimental conditions Click here for file

Additional file 2 **miRNA expressions profile in human embryonic stem cells**. Human embryonic stem cells miRNA expression profile in two experimental conditions Click here for file

Additional file 3 **Novel miRNA candidate expression profiles in human embryonic stem cells**. Using cross-species known miRNAs to construct novel miRNA candidate expression profiles in human embryonic stem cells Click here for file

Additional file 4 **Read number distributions between miRExpress and Fasteris**. Comparison of read number distributions obtained using miRExpress and Fasteris. The correlation coefficients of read number distribution in Mock, BaMV and BaMV+BSF4 are 0.999, 0.998 and 0.996, respectively. Click here for file

Additional file 5 **Read count distributions between miRExpress and Fasteris**. Comparison of read count distributions obtained using miRExpress and Fasteris. Correlation coefficients of read number distribution in Mock, BaMV and BaMV+BSF4 are 0.997, 0.996 and 0.986, respectively. Click here for file

Additional file 6 **Compare miRNA expressions profile in Arabidopsis between miRExpress and aligning reads to genome**. Compare Arabidopsis miRNA expression profile in three experimental conditions by different methods. One is miRExpress. Another is aligning sequence to genome. Click here for file

Additional file 7 **miRNA expressions profile in human embryonic stem cells between miRExpress and Morin et al**. Compare human embryonic stem cell miRNA expression profile in two experimental conditions by different methods. One is miRExpress. Another is Morin et al. Click here for file

Additional file 8 **Spending time in constructing miRNA expression profiles between miRExpress and blast**. The data sets of three experimental conditions in Arabidopsis are used to compare spending time in constructing miRNA expression profiles between miRExpress and blast. Click here for file
