# Supplementary figures and images for: miRExpress: Analyzing high-throughput sequencing data for profiling microRNA expression
Source: BMC Bioinformatics. 2009 Oct 12;10:328. doi: 10.1186/1471-2105-10-328 (PMC2767369; doi:10.1186/1471-2105-10-328)

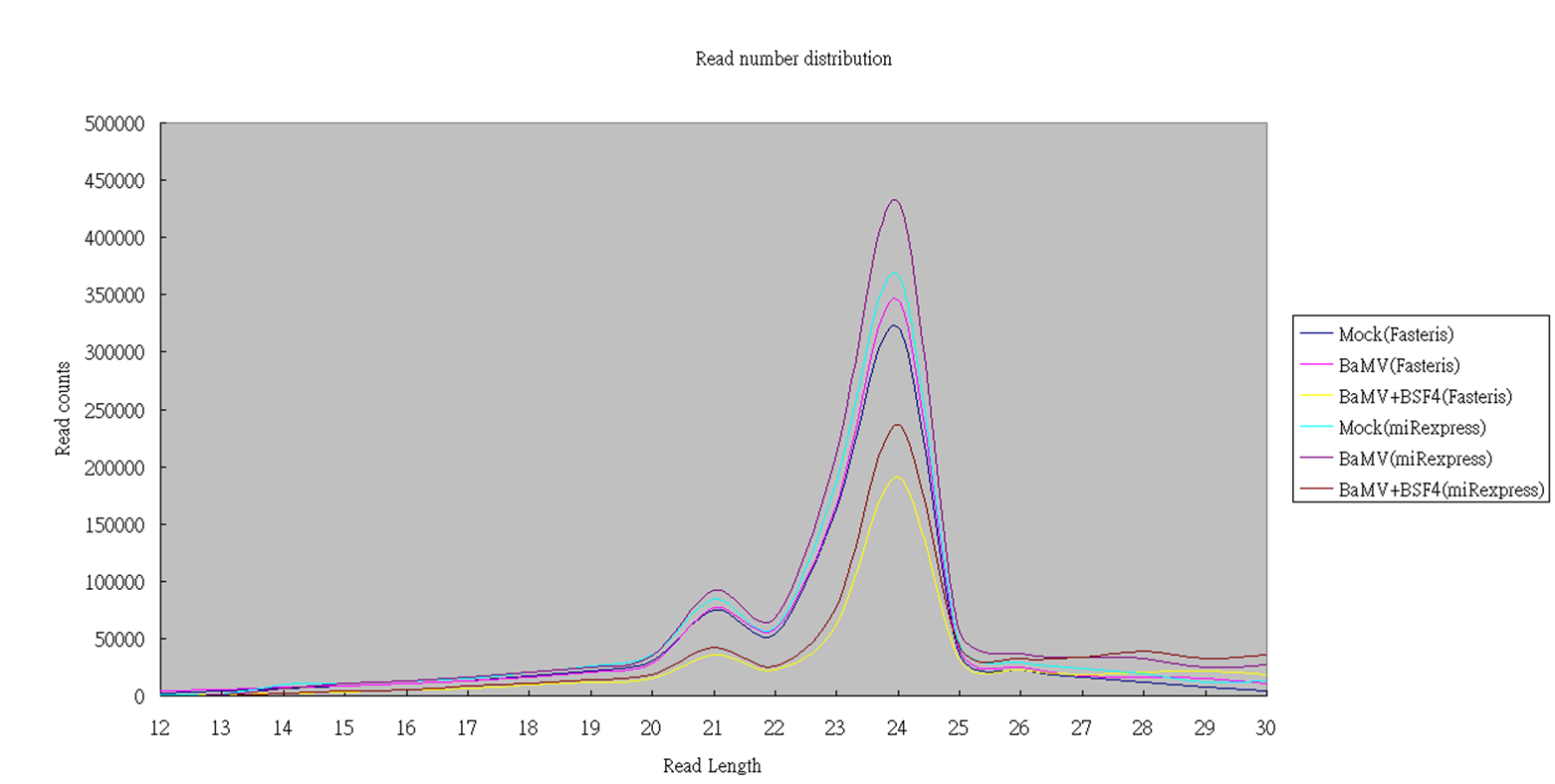

Supplement: Additional file 4 — Read number distributions between miRExpress and Fasteris. Comparison of read number distributions obtained using miRExpress and Fasteris. The correlation coefficients of read number distribution in Mock, BaMV and BaMV+BSF4 are 0.999, 0.998 and 0.996, respectively. [file 1471-2105-10-328-S4.PNG]

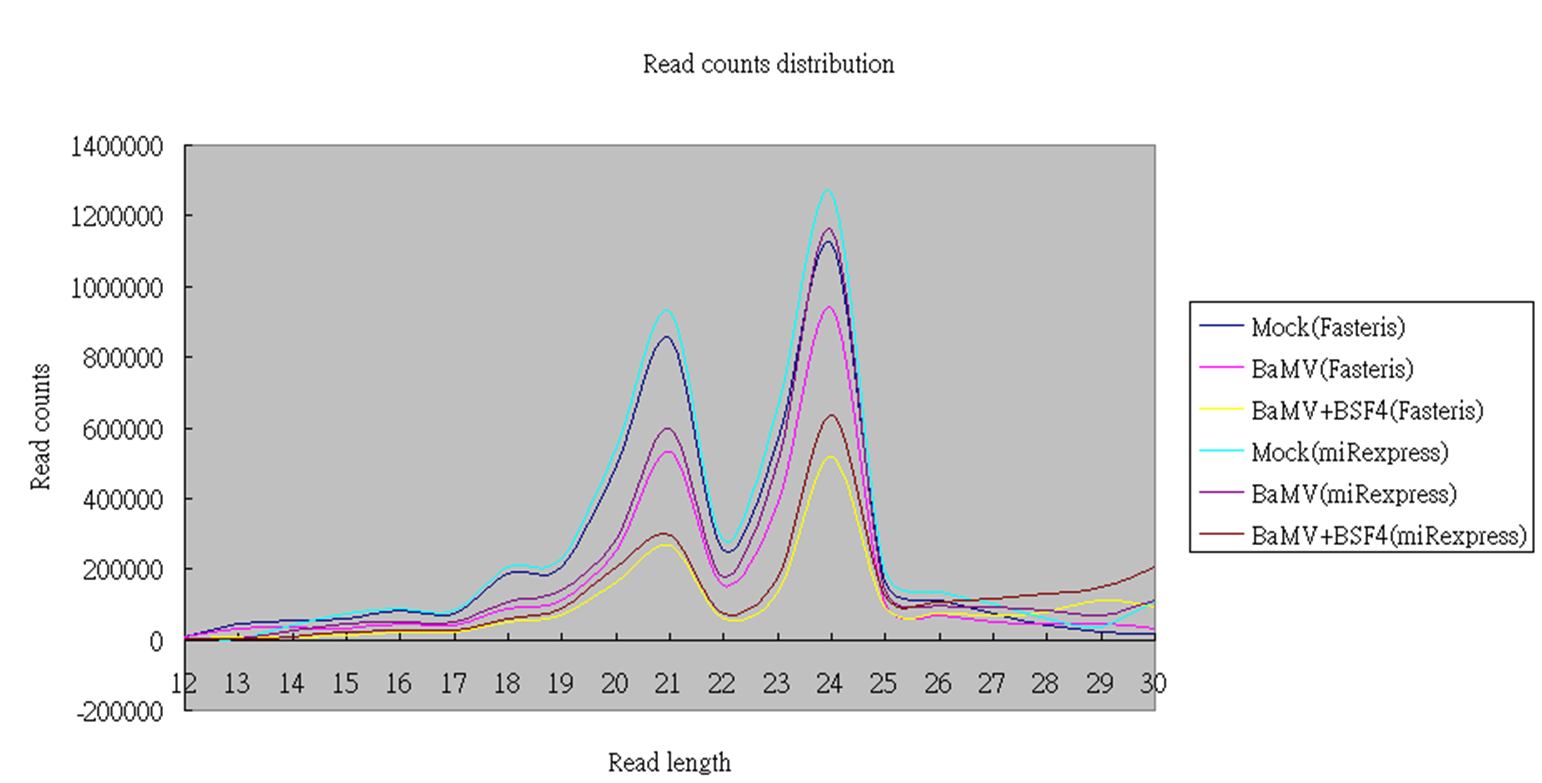

Supplement: Additional file 5 — Read count distributions between miRExpress and Fasteris. Comparison of read count distributions obtained using miRExpress and Fasteris. Correlation coefficients of read number distribution in Mock, BaMV and BaMV+BSF4 are 0.997, 0.996 and 0.986, respectively. [file 1471-2105-10-328-S5.PNG]
